# Norwegian guidelines for persons with concurrent mental disorders and substance use disorders: assessment, treatment and rehabilitation- How to bridge gaps between current practice and clinical guidelines?

**DOI:** 10.1186/1472-6963-14-S2-P67

**Published:** 2014-07-07

**Authors:** Lars Lien, Monica Stolt Pedersen, Anne Landheim

**Affiliations:** 1 National Center for Dual Diagnosis, Hospital Innlandet Trust, Brumunddal, Norway

## Background

There is a lack of experience in implementing clinical guidelines in Norway and most guidelines are put into practice without an implementation plan. This presentation is part of a project dealing with developing strategies for implementing a clinical guideline for persons with concurrent mental disorders and substance use disorders. Purpose To describe the different tools that have been developed to implement the guideline and thereby bridge the gaps between current practice and clinical guidelines.

## Materials and methods

We have put an emphasis on leaders, patients/ families and service providers. For leaders we have established a toolbox for managers including equipment for doing clinical Audits and a practical guide for how to change practice. Representatives from patients organizations have selected and promoted the 10 most important recommendations. An electronic version of the guideline has been developed which in addition to the national dual diagnosis competence training program, are important tools for service providers.

## Results

The national training programme has gathered around 200 to 300 service providers, leaders and users on 17 locations in Norway. Unfortunately, mental health workers and leaders are more absent from the seminars than addiction workers. One important objective of our implementation efforts is that health care workers should participate in continuous training using our internet-based educational training packages like video lectures, instruction videos, screening tools etc. The 10 user selected recommendation have been widely distributed and might also be downloaded as Apps. The clinical Audits have been used in several institutions and departments and we are now developing a community and user Audit. We will present some finding on the use of our net tools and Apps during the presentation.

## Conclusion

In an effort to bridge the GAP between the guideline recommendations and current practice, several measures have been put in place targeting leaders, service providers and users. One of the hurdles we have faced in the implementation is security systems and data programs used by the health authorities making access to our net based tools difficult.

